# Outstanding Reviewers for *Chemical Science* in 2025

**DOI:** 10.1039/d6sc90114j

**Published:** 2026-06-15

## Abstract

We would like to take this opportunity to thank all of *Chemical Science*'s reviewers for helping to preserve quality and integrity in chemical science literature. We would also like to highlight the Outstanding Reviewers for *Chemical Science* in 2025.

## Abstract 2

We would like to take this opportunity to highlight the Outstanding Reviewers for *Chemical Science* in 2025, as selected by the editorial team for their significant contribution to the journal.

## Graphical abstract

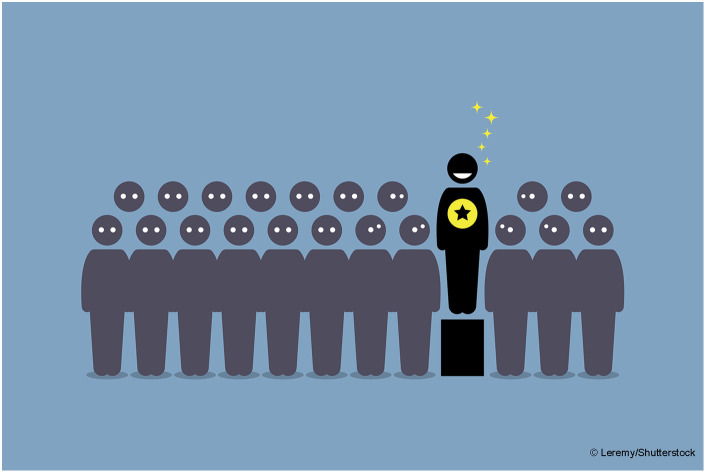

Each one of our outstanding peer reviewers has been carefully selected by our editorial team and includes active researchers who have made significant contributions to peer review and have gone above and beyond in their actions.

As well as selecting reviewers who have provided a higher-than-average number of top-quality reviewer reports, we have also chosen to highlight those reviewers who have provided highly detailed reports and provided constructive feedback, as well as those adjudicative reviewers who have provided thoughtful and robust reports for manuscripts that have initially received conflicting recommendations.

Dr Ana Bahamonde

University of California Riverside

ORCID: 0000-0002-7713-1901

Professor Louise Berben

UC Davis

ORCID: 0000-0001-6461-1829

Dr Alexandra Brumberg

Drexel University

ORCID: 0000-0003-2512-4686

Professor Luca Dell'Amico

University of Padova

ORCID: 0000-0003-0423-9628

Dr Stephen Fielden

University of Birmingham

ORCID: 0000-0001-7883-8135

Dr Renana Gershoni-Poranne

Technion Israel Institute of Technology

ORCID: 0000-0002-2233-6854

Dr Sami Lakhdar

CNRS

ORCID: 0000-0002-1168-7472

Dr Nick Le Brun

University of East Anglia

ORCID: 0000-0001-9780-4061

Dr Dan Li

Jinan University

ORCID: 0000-0002-4936-4599

Dr Felix Plasser

Loughborough University

ORCID: 0000-0003-0751-148X

Dr Liliana Quintanar

Cinvestav

ORCID: 0000-0003-3090-7175

Professor Andrew Sue

Xiamen University

ORCID: 0000-0001-9557-2658

Dr Mercedes Taylor

University of Maryland at College Park

ORCID: 0000-0002-0945-766X

Dr Franziska Thomas

Heidelberg University

ORCID: 0000-0002-1176-7018

Professor Xin-Yao Yu

Anhui University

ORCID: 0000-0003-3576-5815

We would also like to thank the *Chemical Science* Editorial Board and Advisory Board and the wider chemical sciences community for their continued support of the journal, as authors, reviewers and readers.

We continue to work on improving the diversity of our reviewer pool to reflect the diversity of the communities that we serve.

Rebecca Campbell, Executive Editor

